# The missing cost of ecological sleep loss

**DOI:** 10.1093/sleepadvances/zpac036

**Published:** 2022-10-21

**Authors:** John A Lesku, Niels C Rattenborg

**Affiliations:** Sleep Ecophysiology Group, La Trobe University, Melbourne, Australia; Avian Sleep Group, Max Planck Institute for Biological Intelligence, Seewiesen, Germany

## Abstract

Sleep serves many important functions. And yet, emerging studies over the last decade indicate that some species routinely sleep little, or can temporarily restrict their sleep to low levels, seemingly without cost. Taken together, these systems challenge the prevalent view of sleep as an essential state on which waking performance depends. Here, we review diverse case-studies, including elephant matriarchs, post-partum cetaceans, seawater sleeping fur seals, soaring seabirds, birds breeding in the high Arctic, captive cavefish, and sexually aroused fruit flies. We evaluate the likelihood of mechanisms that might allow more sleep than is presently appreciated. But even then, it appears these species are indeed performing well on little sleep. The costs, if any, remain unclear. Either these species have evolved a (yet undescribed) ability to supplant sleep needs, or they endure a (yet undescribed) cost. In both cases, there is urgent need for the study of non-traditional species so we can fully appreciate the extent, causes, and consequences of ecological sleep loss.

**Statement of Significance**

Several recent studies reveal species that routinely sleep little, or can greatly reduce sleep, without any obvious cost to performance, vitality, longevity, or fitness. This stands in contrast to much research showing the daily persistence of sleep in diverse animals, without which, we often see impaired performance. How do we reconcile these incongruent lines of evidence? Are seemingly sleepless animals getting more sleep than is thought? Might adverse effects following extended wakefulness arise from the homeostat trying to enforce sleep? The challenge for the field is to identify the proximate neurological processes that allow some animals to forgo sleep, and to delve deeper into what costs (if any) exist. There is also need for more studies on non-model animals to appreciate the phylogenetic extent of ecological sleep loss.

## Introduction

Converging lines of evidence indicate that sleep serves important functions [1]. Sleep is homeostatically regulated such that sleep lost through extending wakefulness is recovered by sleeping more and/or more intensely [2, 3]. Sleeping animals are vulnerable to predators and rivals owing to reduced environmental awareness and slowed behavioral responsiveness [4]. Nonetheless, despite these dangers, sleep is found across the animal kingdom, including some of the least complex animals [5–8]. Lastly, losing sleep is (typically) followed by impaired waking performance [9–12]. Consequently, the indispensability-of-sleep is a long-standing pillar of sleep science.

The amount of sleep an animal obtains reflects species-specific trade-offs weighing the costs of sleep (e.g. heightened risk, missed opportunities) against its benefits (e.g. synaptic scaling, glymphatic clearance of metabolic waste, energy homeostasis). When the costs of sleeping exceed the benefits, wakefulness is favored. But sustained wakefulness is itself unsustainable owing to high energetic costs [13, 14], the accumulation of neural waste [15], unchecked synaptic potentiation [16], and performance impairments [9–12]. And so, an optimal solution must be reached. In this way, the great interspecific variation observed in the timing, duration, and composition of sleep reflects the optimal sleep architecture for that species in that environment. For example, large herbivorous mammals sleep very little in barns, zoos, and in the wild, just a handful of hours per 24-h day [17–21]. Short-sleeping mammals favor being awake, perhaps because their nutritionally impoverished diet demands voluminous consumption achieved through time-intensive foraging [22]. Other species show remarkable plasticity in the amount of sleep, such as songbirds that sleep 5 h more in winter [23, 24]. The question remains of *how* these animals are able to perform adaptively on little sleep.

Even as research underscoring the need for sleep continues to emerge, so too does new and mounting evidence of animals that forgo sleep without obvious costs. Perhaps not surprisingly, such studies gravitate towards species living in (what we might call) extreme environments. Bias of this sort is common in comparative physiology since these species often best elucidate physiological principles. That is, when animals are pushed to extremes, the nature and range of adaptations is revealed, while they remain more subtle in moderate environments. As such, we discuss studies on swimming cetaceans, soaring seabirds, and others. When pertinent, we evaluate mechanisms that may covertly permit sleep to sustain adaptive waking performance. However, it becomes clear that much research remains to understand the specific costs—if any—associated with extreme sleep loss in this growing list of case-studies.

## Elephant Matriarchs

**Figure 1.**
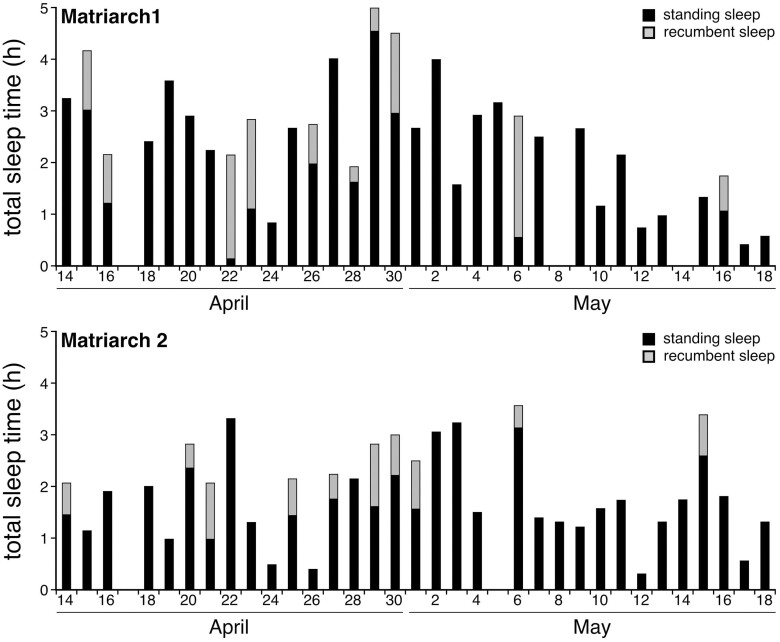
The amount of sleep, defined as sustained (≥ 5 min) trunk immobility, in two African bush elephant matriarchs over 5 weeks. Elephants could sleep while standing (black) and recumbent (grey); on some days, the elephants appeared to go without any sleep. Reprinted from Gravett et al. (2017) African elephant matriarchs: does large body size make elephants the shortest mammalian sleepers? *PLOS ONE* 12: e0171903.

For a half-century it has been known that bigger mammals sleep less [20, 22, 25]. A recent study on the African bush elephant (*Loxodonta africana*) shows that the largest terrestrial mammal is no exception [21]. Two elephant matriarchs received trunk-implanted accelerometers and the animals were released back into the Chobe National Park in Botswana for a five-week recording of activity. In the absence of electroencephalographic (EEG) measures of sleep, Gravett and colleagues [21] considered trunk immobility (5 min and longer) to be a behavioral indicator of sleep (see also [18]). Using this criterion, the elephants slept an average of 2 h per 24-h day [21]; 2–3 times less than that reported for captive, female Asian elephants (*Elephas maximus*) video recorded for 10 months [18]. Most sleep occurred while the animals were standing, similar to behavioral work on giraffes (*Giraffa camelopardalis*) [19], and EEG-based research on horses (*Equus ferus caballus*) and sheep (*Ovis aries*) [17]. The African bush elephants laid down to sleep only every third or fourth day. Such prolonged standing may curtail the opportunity for rapid eye movement (REM) sleep and its associated reduction of skeletal muscle tone. Even more interestingly, the elephants did not appear to sleep every day. On multiple occasions, the elephants were ostensibly sleepless for 45–48 h (Figure 1). In humans, extending the waking day from 16 to 20 h reduces performance on a cognitive psychomotor test to the level of someone legally intoxicated (blood alcohol concentration of 0.05%) [9]. How then are elephants able to perform well, in the wild, while routinely sleeping so little?

One possibility is that the elephants were sleeping more than was thought. While we appreciate the ethical questions and logistical challenges of recording EEG signals through the 30 cm-thick cranium of adult elephants, the use of trunk immobility as a proxy for sleep is unverified (as noted by Gravett and colleagues [21]). As such, the elephants may have fallen asleep rapidly between trunk movements, much in the way that dusky antechinus (*Antechinus swainsonii*) (a small marsupial mammal) falls asleep quickly once immobile and has hundreds of sleep bouts per day, each mere tens of seconds long [26]. Furthermore, the elephants could have spent considerable amounts of time ‘drowsy’. Drowsiness, while inconsistently defined in comparative sleep research, combines aspects of wakefulness (e.g. activated EEG) and non-REM sleep (e.g. slow-waves) [27]. Many farm animals spend a lot of time in this mixed state; during the night, cows (*Bos taurus*) spend more time drowsy than they do alertly awake, or in non-REM and REM sleep [17]. It is possible that elephants, too, have recurring slow-waves over a background of waking EEG activity that might (cumulatively) reflect more non-REM sleep than was appreciated. That said, elephants would likely still have had much less sleep than most mammals, and go without REM sleep for days. Accordingly, it is also possible that elephants simply endure the physiological and performance costs of extended wakefulness.

## Sea Mammals

Mammals are a derived type of therapsid, and first appeared in the late-Triassic more than 200 million years ago. Most of these early mammals scurried on the ground, while some could glide amongst the tree canopy [28]. After the extinction of all non-avian dinosaurs at the end of the Cretaceous, the mammalian lineage diversified rapidly to occupy the vacant ecological niché once filled by reptiles. Fifty million years ago, three mammalian lineages (cetaceans, sirenians, and pinnipeds) independently embarked on the evolutionary invasion of the sea. They faced an immediate challenge of breathing atmospheric oxygen while sleeping in a marine environment. The response was the convergent evolution of an elegant solution: the ability to sleep with one-half of the brain, or unihemispheric non-REM sleep [29].

For cetaceans (whales and dolphins), unihemispheric non-REM sleep is the dominant form of sleep and affords the abilities of continuous swimming (to position the blowhole above the water’s surface) and vigilance (via the eye and ear contralateral to the awake hemisphere) [29]. Convincing electrophysiological evidence for REM sleep in cetaceans is lacking. That said, behavioral observations of cetaceans reveal wholly immobile postures that may reflect either bihemispheric non-REM sleep or REM sleep [30–33]. Like cetaceans, sirenians (manatees, *Trichechus* spp.) and some pinnipeds (otariid seals and walrus, *Odobenus rosmarus*) also engage in unihemispheric non-REM sleep, but unlike cetaceans, show unequivocal REM sleep and (bihemispheric) non-REM sleep; phocid seals maintain the strictly bihemispheric sleep of their terrestrially sleeping ancestors [29].

In cetaceans, we find examples of great plasticity in the amount of sleep, seemingly without detriment. For four months, Lyamin and colleagues [31] observed swimming behaviour in two, captive, pregnant killer whale (*Orcinus orca*) cows, and continued their observations 5 months after parturition. Alone, a pregnant orca cow will spend 5–8 h per 24-h day ostensibly asleep, during which they float, motionless, at the surface, or lay immobile on the bottom of the pool. This is in marked contrast to the near continuous swimming observed in the cow, and also the calf, in the weeks post-partum. While both cow and calf increased the amount of time spent restful over weeks and months, their levels never equaled that of the pregnant cow. Moreover, during the weeks of ostensible sleeplessness, the patterns of swimming were dynamic. The animals actively avoided obstacles in the pool, including walls and other animals; collisions were never observed. Lyamin and colleagues [31] also observed four bottlenose dolphin (*Tursiops truncates*) cows and their calves and found a similar pattern to the killer whale pairs. Their observations are in agreement with work on bottlenose dolphins that maintain auditory responsiveness for days [34].

How do killer whale and bottlenose dolphin cows and calves perform well, seemingly with so little sleep? In other species, the youngest animals sleep the most [35]. This conserved ontogenetic pattern is thought to reflect a need for sleep (particularly REM sleep) to facilitate maturation of the developing nervous system [36, 37]. Whether cetaceans engage in REM sleep is equivocal [29], yet continuous swimming of calves would, in any case, appear to be incompatible with REM sleep. Given their precocial nature, perhaps calves engage in REM sleep *in utero*, not unlike the high proportion of REM sleep observed in prenatal lambs [38]. REM sleep aside, cows and calves still have little outward restfulness in the weeks and months after birth. It is likely that the cetaceans, including the calves, were engaging in unihemispheric non-REM sleep. Although both eyes were almost always open when the animals surfaced to breathe [31], they spend considerable time underwater with one eye closed [39]. In this way, mother and offspring obtain at least some sleep. Nonetheless, whether this amount of unihemispheric sleep is equivalent to the amount of sleep they get when they are older and motionless is unclear.

**Figure 2.**
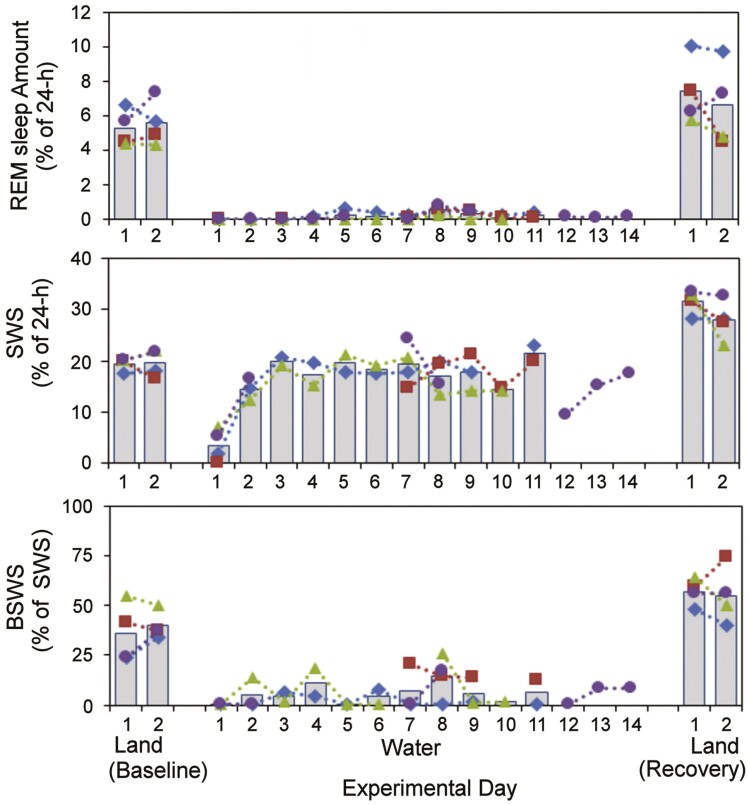
The amount of rapid eye movement (REM) sleep (*top*) and slow-wave sleep (SWS), with the latter including bihemispheric (BSWS) and unihemispheric SWS (*middle*), and the proportion of SWS that was BSWS (*bottom*) in northern fur seals when the animals slept on land (*baseline*), in seawater, and back on land (*recovery*). REM sleep was substantially reduced during the two-week period in seawater, without evidence for a REM sleep rebound during recovery. Conversely, the seals achieved a daily amount of SWS in seawater by sleeping unihemispherically. Colored lines and symbols denote individual seals. Modified from Lyamin et al. (2018). Fur seals suppress REM sleep for very long periods without subsequent rebound. *Curr Biol.* 28:2000–2005.

Otariid seals pose another challenge to the adaptive value of REM sleep. Unlike wholly aquatic cetaceans, seals may sleep either in the sea or on the land. While on land, Lyamin and colleagues [40] found that the northern fur seal (*Callorhinus ursinus*) engages predominantly in bihemispheric non-REM sleep and obtains 80 min of REM sleep per 24-h day. When forced to sleep in seawater for two weeks, the seals switch to unihemispheric non-REM sleep and the amount of REM sleep drops to just 3 min per day (Figure 2). Furthermore, no REM sleep was observed during the first week (days 3–7) in seawater. By the end of their 10th landless day, the seals had accumulated a 13 h REM sleep deficit. When allowed to sleep on land again, the seals recovered only a small fraction of this amount. Thus, the majority of REM sleep lost was never recovered. This extreme result in fur seals is inconsistent with considerable data from diverse species that show REM sleep rebound following sleep loss. Nevertheless, the result is consistent with a handful of studies that report either no, or only modest, REM sleep rebound [41–43]. The cause and consequence of seemingly unregulated REM sleep is unclear.

## Avian Dinosaurs

**Figure 3.**
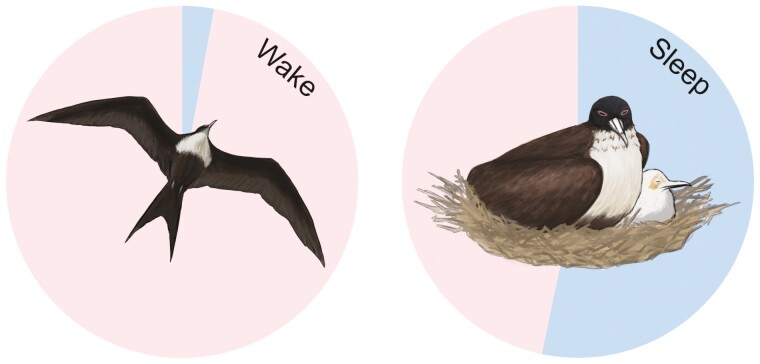
The amount of wakefulness (*red*) and sleep (*blue*) in great frigatebirds on-the-wing (*left*) and nesting (*right*). While flying, frigatebirds sleep one-eighteenth what they achieve on land. Data extracted from Rattenborg et al. (2016) Evidence that birds sleep in mid-flight. *Nat Commun* 7: 12468 and reprinted from Lesku and Schmidt (2022). Energetic costs and benefits of sleep. *Curr Biol* 32: R656-R661; illustrations by Laura X. Tan.

Some birds are capable of remarkable feats of flying that impinge upon achieving a daily amount of sleep [44]. Notably, the great frigatebird (*Fregata minor*) is a large seabird that soars over the ocean in pursuit of sub-surface prey. Unfortunately for frigatebirds, they cannot safely alight on the water owing to short legs, poorly developed foot webbing, and inadequate waterproofing. As such, they rely on oceanic predators to drive prey, such as flying fish and squid, near or above the surface of the sea where they become accessible to the frigatebirds. This ephemeral food resource requires the birds to travel long distances over the ocean. A multi-year study into the large-scale movements of great frigatebirds found that the birds were adept at using favorable winds and convection in order to fly for two months, non-stop, during transoceanic flights [45]. Rattenborg and colleagues [46] provided the first electrophysiological evidence that frigatebirds, and indeed any bird, can sleep while flying. Equipped with a head-mounted accelerometer, back-mounted GPS, and implanted with electrodes for recording brain activity, the birds were found to engage in bihemispheric and unihemispheric non-REM sleep, and REM sleep, in flight. While impressive, the frigatebirds were also remarkable for how little they slept, just 0.7 h per 24-h day (Figure 3). The sleep obtained aloft was also more fragmented, less intense (based on EEG slow-wave activity), and more unihemispheric than observed on land. Such restricted and fragmented sleep is not a fixed feature of frigatebird biology, however, because when back on the nest, the birds slept nearly 13 h per day. Therefore, great frigatebirds can subsist on very little sleep for weeks, and likely months, while maintaining the cognitive abilities necessary to track thermals, and find and consume prey.

Like many birds, some shorebirds are migratory. Notably, the pectoral sandpiper (*Calidris melanotos*) migrates each spring from the southern hemisphere to above the Arctic Circle to breed under continuous daylight. Once the birds arrive, males set up territories, which they defend against rival males through ground and aerial displays, competitive flights, and physical fights. Males also display to females with whom they want to mate. Because pectoral sandpipers are polygynous and male investment into reproduction is limited to copulation, males are motivated to simply mate with as many females as possible. Conversely, female investment is high: the energetic demands of egg production, time-intensive incubation, and post-hatching parental care to supervise the precocial chicks. As such, unlike the indiscriminate males, females scrutinize males to find the best to father her only clutch of the season.

**Figure 4.**
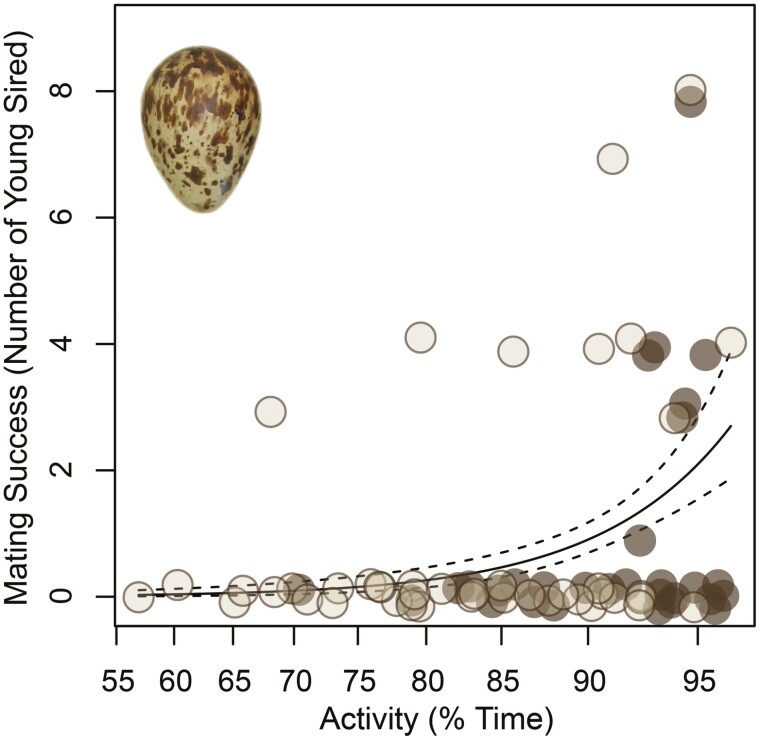
Male pectoral sandpipers vary in their level of activity during the breeding season. Some males are incredibly active, which corresponds with a profoundly low amount of sleep. Nonetheless, the level of activity predicts the number of offspring a male sired in a given year. Datapoints are raw values from one of two years (*tan*, *brown*); the fitted line includes the 95% confidence interval (*dashed*). Reprinted from Lesku et al. (2012). Adaptive sleep loss in polygynous pectoral sandpipers. *Science* 337: 1654-1658.

In a multi-year study, Lesku and colleagues [47] recorded the activity levels of male and female sandpipers on the tundra, interactions between the sexes, genetically-resolved paternity of most chicks on the study site, and, for a subset of males, brain activity to measure the amount and intensity of sleep. In doing so, they demonstrated that males have the highest activity levels during the mating period. Some males were extremely active—upwards of 95% of the time for three weeks. Not all males were able to achieve, or sustain, this high level of activity, however. At this point in the study, it remained unclear whether males varied in the amount of restful wakefulness or sleep *per se*. To distinguish between these alternatives, recordings of brain activity of males on the tundra revealed that the relatively restful males were sleeping much more than the extreme males, who indeed slept very little—packaged into hundreds of short sleep bouts per 24-h day. Extremely active males maintained a need for sleep as reflected in high non-REM sleep slow-wave activity during those micronaps. And yet, their attempt to recover lost sleep by sleeping more intensely was unsuccessful, because the cumulative amount of slow-wave activity (or slow-wave energy) was no greater in the short-sleeping males. While these males slept little, and retained a sleep debt, they nonetheless interacted with the most females, convinced females to mate with them, and ultimately sired the most chicks (Figure 4). This study constituted the first direct evidence that sleep loss can be evolutionarily adaptive, but it remains unclear how some males were able to perform so well on so little sleep.

## Captive Cavefish

**Figure 5.**
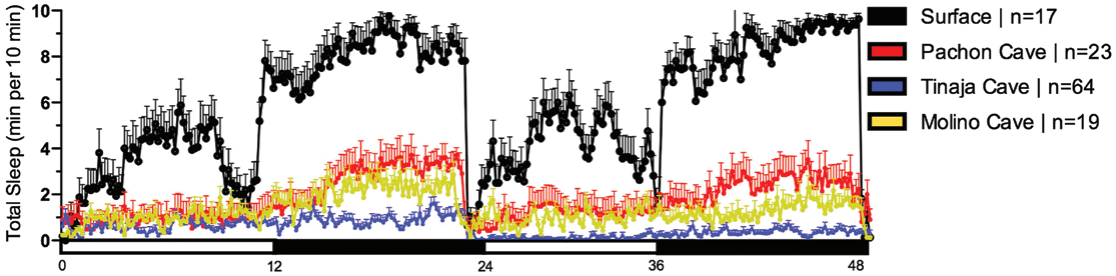
The amount of sleep in one captive population of river-living Mexican tetras (*surface*) and three cave-dwelling populations (*Pachón*, *Tinaja*, *Molino*) over 48-h. Surface fish sleep a lot and predominantly at night, whereas the cavefish (1) sleep very little and (2) do not have strong circadian rhythmicity. Reprinted from Duboué et al. (2011). Evolutionary convergence on sleep loss in cavefish populations. *Curr Biol* 21:671–676.

Thus far we have discussed cases of sleep plasticity in response to changing environmental conditions: sleeping in the water, in the air, and during the breeding season. However, comparing sleep between populations is also fruitful. The Mexican tetra (*Astyanax mexicanus*) is a small freshwater fish. They are noteworthy because populations of this species have independently invaded, and evolved to live in, perpetually dark caves. In doing so, the cave-dwelling populations have lost pigmentation, but also their eyes, in marked contrast to their river-living siblings. Duboué and colleagues [48] compared the amount of sleep between surface and cave populations. Cavefish (and indeed other fish) sleep, which in these tetras can be characterized behaviorally as restfulness lasting 60 s or longer, and corresponding to reduced responsiveness (see also [49–51]). Interestingly, captive river-living tetras were found to sleep 13.3 h per 24-h day, yet tetras from three populations living in caves slept just 1.8–4.2 h (Figure 5). Why do subterranean fish sleep less than members of the same species living on the surface? There is some evidence that the neuropeptide hypocretin (orexin) is involved with sleep loss in cavefish [52]. Energetics may also play a role since food restriction induces sleep [53]. Perhaps the simplified cave environment (relative to the rich heterogeneity of environmental variation found in river systems) impedes neurobiological changes (e.g. synaptic potentiation, metabolic waste build-up) that decrease the need for sleep. Lastly, it is also important to understand how these fish sleep in the wild, given that all measurements were made under a 12-h light/ 12-h dark photoperiod. Such cycles are peculiar to cavefish. Despite degeneration of both eyes during embryonic development, larval cavefish remain responsive to light [54]. So too do adults given the activity of cavefish increased during the light phase [48].

## Fruit Flies

Case-studies of animals with reduced sleep are not limited to charismatic megafauna, extreme birds, or unusual fish. Under some circumstances, the humble fruit fly (*Drosophila melanogaster*) can lose their ability to recover sleep lost through stimulating encounters while awake. Beckwith and colleagues [55] motivated male flies to stay awake through interactions with other male flies. Once these tired males were socially isolated and allowed to sleep, they recovered lost sleep by sleeping longer. Not unlike male pectoral sandpipers [47], male fruit flies also lose sleep in the presence of receptive females. However, the male does not recover lost sleep after the female has been removed. In addition, the female *per se* is not necessary to elicit this peculiar lack-of-response, rather only her pheromones. And so, it is thought that sexual arousal, induced by female aromatic cues, stimulates wakefulness in males and overrides the sleep homeostat. Consistent with this interpretation, Beckwith and colleagues [55] found that males insensitive to female cues (via the absence of the pheromone receptor), show a typical sleep rebound. This response has been linked to the activity of a cluster of neurons that, when stimulated, inhibit sleep homeostasis [55–57]. Given the recovery of lost sleep evinces the essential nature of sleep, unregulated sleep in flies might suggest that some sleep does not serve a vital function.

**Figure 6.**
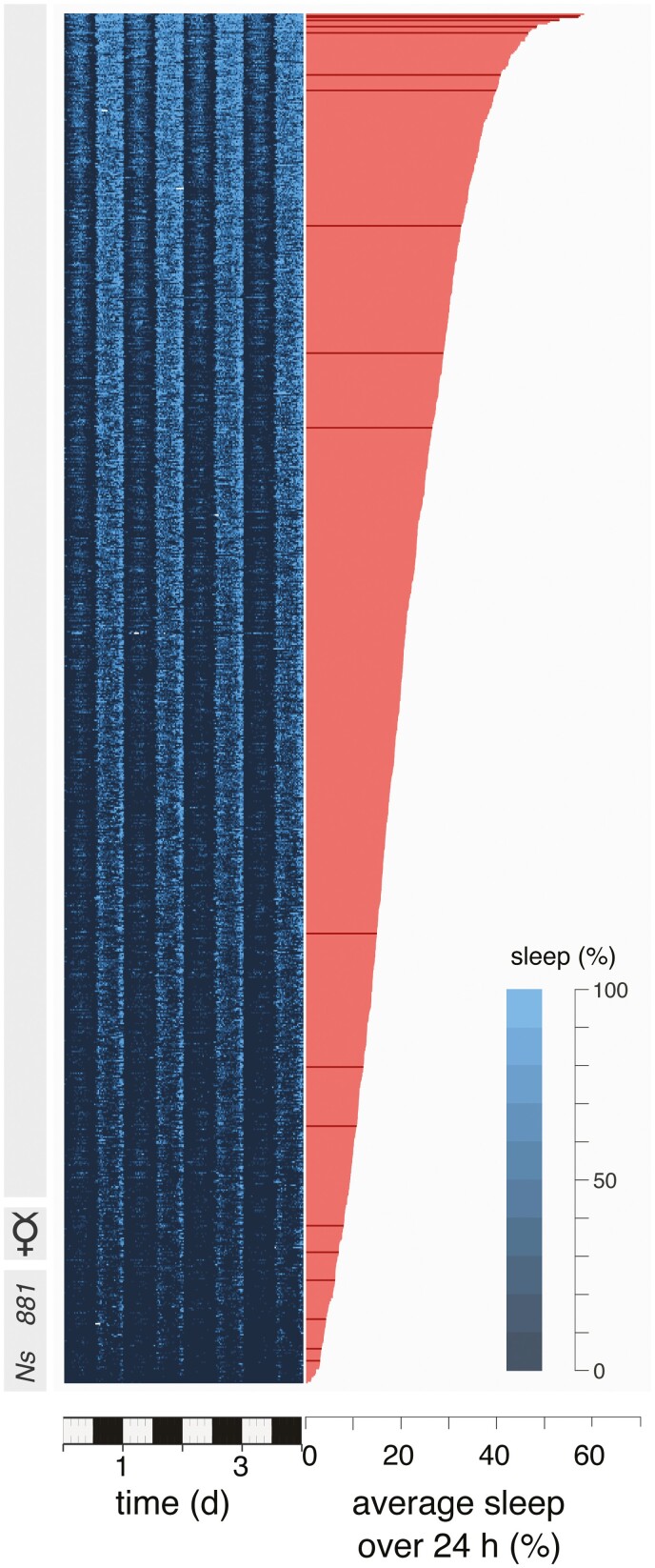
Sorted sleep amounts for 881 individual female fruit flies from most (*top*) to least (*bottom*) sleep over 4-d (*left*) and as a 24-h mean (*right*), revealing great intraspecific variation in the amount of sleep in a wild-type *Drosophila* strain and apparent sleeplessness of some individuals. Modified from Geissmann et al. (2019). Most sleep does not serve a vital function: evidence from *Drosophila melanogaster*. *Sci Adv* 5: eaau9253.

Geissmann and colleagues [58] provide tantalizing hints to this end. Using a high-throughput system for analyzing movements from large numbers of wild-type flies housed individually in tubes, they found that most male flies sleep *circa* 10.3 h. Conversely, females routinely slept much less: 5.0 h per day. Interestingly, 6% of the 881 females studied, slept less than 1.2 h. The three most extreme females slept just 4–15 min per 24-h day (Figure 6). To determine whether short-sleeping females have shorter longevity, Geissmann and colleagues [58] stimulated sleeping flies to move for the duration of their lives. The intended sleep deprivation was achieved by rotating the housing-tube five times over 1 s after the fly had been restful for 20 s. Surprisingly, no effects on male survival were noted; females showed only a 9% reduction to life span. Thus, in wild-type fruit flies, endogenously driven, and artificially induced, sleep loss does not affect longevity. That said, the reliance on behaviour alone to characterize sleep, and the long time-interval to detect sleep, could have allowed the flies to sleep more than is appreciated. Early in the sleep deprivation procedure, the flies could have started to package their sleep in 20 s bouts. As sleep pressure grew, the flies may also have continued to sleep during the tube rotation. Furthermore, flies can move during sleep and wakefulness; to distinguish these states one should measure arousal thresholds, if not also local field potential power [59]. For these cautionary reasons, the significance of this study is unknown. Nonetheless, it does raise the intriguing possibility that some sleep may be non-vital.

## Mitigating Mechanisms?

Armed with this growing catalogue of examples, three possible conclusions emerge. One, these animals are covertly sleeping, and by doing so, getting more sleep than is thought. Two, the animals do indeed sleep little, but endure the neurological and/or physiological costs. Three, reported sleep times are accurate and the species considered have evolved (yet unknown) adaptations to thrive on little sleep. In the text above, we considered drowsiness (elephants), unihemispheric non-REM sleep (cetaceans), and motivational shifts and micronaps (flies) as mechanisms that might mitigate against performance decrements while awake. What other means might animals have to achieve the functional benefits of sleep while awake?

Given sleep typically precludes activities often associated with being awake, a state that combines sleep and wakefulness might seem an effective evolutionary strategy. Using a mathematical model, Lima and Rattenborg [60] provided the argument that such a state, were it found to exist, would be maladaptive, owing to the interdependency of neural components. This prediction later found some empirical support. Vyazovskiy and colleagues [61] first characterized the phenomenon of local sleep in an otherwise behaviorally awake animal. Rats were implanted with subcortical electrodes in the motor and parietal cortices. Local electrophysiological signs of non-REM sleep could appear while the rats were behaviorally awake and responsive to stimuli. As sleep pressure accrued across a 4-h sleep deprivation, the occurrence of local non-REM sleep increased, suggesting that it reflects the homeostatic pressure to recover lost sleep. Interestingly, when presented with a motor task, rats performed worse when local sleep occurred in the motor, but not parietal, cortex. Similar lapses have been reported in locally sleeping humans performing a psychomotor vigilance task [62]. Thus, as predicted [60], there is a performance cost to engaging in local sleep in rodents and humans. Consequently, this phenomenon is unlikely to explain high, sustained performance in great frigatebirds and pectoral sandpipers wherein some individuals are extremely active, around-the-clock, for weeks. Recent technological advances should permit the study of depth local field potentials across many brain regions from freely-behaving animals in the wild to look for signs of local sleep [63].

## Conclusions

And so, we are left with a paradox. We have the daily persistence and evolutionary stubbornness of sleep, without which, we often see impaired performance in diverse taxa. And we have an emerging cohort of species that routinely sleep little, or can greatly reduce sleep, without any obvious cost to performance, vitality, longevity, or fitness. How do we reconcile these disparate lines of evidence?

Some adverse effects we see following sleep loss might arise from the homeostat trying to enforce sleep. The regulation of *eating* can be a helpful analogy for the regulation of sleep. Eating homeostasis ensures that the organism maintains a regular (often daily) intake of calories. When energy stores drop, the eating-homeostat makes an animal hungry and, in doing so, motivated to eat. If an animal skips a meal, then it will eat more, and more intensely, when presented with food. Importantly, the animal does not suffer obvious behavioral and physiological impairments by missing one, or perhaps a few, meals. Instead, the eating-homeostat serves a protective role to prevent the accumulation of an energy deficit, which, when substantial, will cause impaired performance. The sleep homeostat serves a similar protective role—to prevent the accumulation of maladies that sleep otherwise remedies. The inattention and reduced motivation observed in tired animals might reflect an insistent sleep homeostat [12]. Fruit flies can turn-off or otherwise overwhelm the sleep homeostat through competing demands, such as sexual arousal [55]. In such cases, are there long-term costs to blocking sleep homeostasis? Possible costs might include surging energy consumption [13, 14], unchecked synaptic potentiation [16], shortened lifespan or curtailed lifetime reproductive success. The immediate challenge is to identify the proximate neurological processes that allow some animals to forgo sleep, and to delve deeper into what costs may exist, but had been overlooked.

Lastly, there is an urgent need for the study of non-traditional animals. Much of our understanding of sleep comes from a handful of species, notably humans, laboratory rodents, and fruit flies. This bias is not unique to sleep research, but rather is common to neuroscience and physiology [64]. As such, we need a proliferation of studies into comparative animal sleep research that embraces the full diversity of sleep seen across the animal kingdom [8, 65–67]. Only through such an ambitious enterprise can we appreciate the extent, causes, and consequences of ecological sleep loss.

## Notes

Financial disclosure: None.

Non-financial disclosure: None.
